# Transcriptome profiling of two contrasting ornamental cabbage (*Brassica oleracea var. acephala*) lines provides insights into purple and white inner leaf pigmentation

**DOI:** 10.1186/s12864-018-5199-3

**Published:** 2018-11-06

**Authors:** Si-Won Jin, Md Abdur Rahim, Khandker Shazia Afrin, Jong-In Park, Jong-Goo Kang, Ill-Sup Nou

**Affiliations:** 1 Department of Horticulture, Sunchon National University, Suncheon, 57922 Republic of Korea; 2 Department of Genetics and Plant Breeding, Sher-e-Bangla Agricultural University, Dhaka-1207, Bangladesh

**Keywords:** Transcriptome, Ornamental cabbage, Anthocyanin biosynthesis, Chlorophyll biosynthesis, Leaf color

## Abstract

**Background:**

Ornamental cabbage (*Brassica oleracea var. acephala*) is an attractive landscape plant that remains colorful at low temperatures during winter. Its key feature is its inner leaf coloration, which can include red, pink, lavender, blue, violet and white. Some ornamental cabbages exhibit variation in leaf color pattern linked to leaf developmental stage. However, little is known about the molecular mechanism underlying changes in leaf pigmentation pattern between developmental stages.

**Results:**

The transcriptomes of six ornamental cabbage leaf samples were obtained using Illumina sequencing technology. A total of 339.75 million high-quality clean reads were assembled into 46,744 transcripts and 46,744 unigenes. Furthermore, 12,771 genes differentially expressed across the different lines and stages were identified by pairwise comparison. We identified 74 and 13 unigenes as differentially expressed genes related to the anthocyanin biosynthetic pathway and chlorophyll metabolism, respectively. Among them, three unigenes (*BoC4H2*, *BoUGT9*, and *BoGST21*) and six unigenes (*BoHEMA1*, *BoCRD1*, *BoPORC1*, *BoPORC2*, *BoCAO*, and *BoCLH1*) were found as candidates for the genes encoding enzymes in the anthocyanin biosynthetic pathway and chlorophyll metabolism, respectively. In addition, two unigenes (*BoRAX3* and *BoTRB1*) as MYB candidates, two unigenes (*BoMUTE1,* and *BHLH168-like*) as bHLH candidates were identified for purple pigmentation in ornamental cabbage.

**Conclusion:**

Our results indicate that the purple inner leaves of purple ornamental cabbage result from a high level of anthocyanin biosynthesis, a high level of chlorophyll degradation and an extremely low level of chlorophyll biosynthesis, whereas the bicolor (purple/green) outer leaves are due to a moderate level of anthocyanin biosynthesis, a high level of chlorophyll degradation and a very low level of chlorophyll biosynthesis. In white ornamental cabbage, the white inner leaves are due to an extremely low level or absence of anthocyanin biosynthesis, a high level of chlorophyll degradation and a very low level of chlorophyll biosynthesis, whereas the bicolor (white/green) leaves are due to a high level of chlorophyll degradation and a low level of chlorophyll biosynthesis and absence of anthocyanin biosynthesis. These results provide insight into the molecular mechanisms underlying inner and bicolor leaf pigmentation in ornamental cabbage and offer a platform for assessing related ornamental species.

**Electronic supplementary material:**

The online version of this article (10.1186/s12864-018-5199-3) contains supplementary material, which is available to authorized users.

## Background

Ornamental cabbage (*Brassica oleracea var. acephala*) is an attractive landscape plant valued for its fascinating inner leaf coloration and its ability to grow and remain colorful at temperature as low as 15–20 °F [1, 2]. It is becoming increasingly well-known for its long-lasting, colorful leaves and hardiness during fall and early winter when many plants senesce. The characteristic feature of ornamental cabbage is the inner leaf colors, which can include red, pink, lavender, blue, violet and white [2]. Some ornamental cabbages exhibit variation in color pattern in the leaves along with leaf developmental stages.

This spatiotemporal variation in leaf pigmentation is due to the accumulation of anthocyanins [2, 3]. Across plant species, anthocyanin accumulation is more frequent at juvenile stages, generally causing reddening of young leaves and degrading during maturation (reviewed by Oren-Shamir [4]). In juvenile plants, anthocyanins protect leaves from damage caused by UV irradiation, photoinhibition and oxidative damage. In mature plants, however, leaves accumulate increasing amounts of chlorophyll and wax compounds that also provide photoprotection, and therefore alter their color from red to green through degradation of anthocyanins (reviewed by Oren-Shamir [4]).

Anthocyanins are the largest groups of plant pigments and the determining factor for red, purple, violet and blue pigmentation of different plant parts, including leaves, stems, roots, flowers and fruits [2, 3, 5–8]. They are biosynthesized through the flavonoid branch of the phenylpropanoid biosynthetic pathway [9, 10]. The anthocyanin biosynthetic pathway is one of the best characterized pathways in plants [7, 11–13]. The structural and regulatory genes of anthocyanin biosynthesis are well characterized in *B. oleracea* subspecies, including ornamental cabbage [3, 6, 14]. The structural genes of the anthocyanin biosynthetic pathway are *phenylalanine ammonia-lyase* (*PAL*), *cinnamate 4-hydroxylase* (*C4H*), *4-coumaroyl CoA-ligase* (*4CL*), *chalcone synthase* (*CHS*), *chalcone isomerase* (*CHI*), *flavanone 3-hydroxylase* (*F3H*), *flavonoid 3′-hydroxylase* (*F3′H*), *dihydroflavonol 4-reductase* (*DFR*), *leucoanthocyanidin dioxygenase* (LDOX), *UDP-flavonoid glucosyl transferase* (*UFGT*) and *glutathione S-transferase* (*GST*) [5, 9, 15, 16]. Meanwhile, the regulatory genes of the anthocyanin pathway encode proteins in the myeloblastosis (MYB), basic helix-loop-helix (bHLH) and WD40 repeat (WDR) transcription factor (TF) families [7, 17–20]. These TFs form a MBW complex and coordinately regulate the transcription of structural genes [15, 21].

Chlorophyll is vital for solar light harvesting and energy transport to the reaction centers during photosynthesis [22]. Chlorophyll biosynthesis is a complex process catalyzed by more than 17 enzymes [23]. The genes involved in the chlorophyll metabolic pathway in angiosperms have been identified and characterized (reviewed from Beale [24]). The chlorophyll biosynthesis pathway has three different steps: synthesis of chlorophyll *a* from glutamic acid*,* interconversion of chlorophyll *a* and chlorophyll *b*, and degradation of chlorophyll *a* into colorless linear tetrapyrroles [25–27]. To date, the molecular mechanism of chlorophyll metabolism in ornamental cabbage has not been characterized, and the changes in leaf pigmentation that occur in the course of development remain unclear.

Here, we performed transcriptome profiling of samples from three different stages of leaf development from purple and white ornamental cabbage lines using the Illumina sequencing platform, focusing on the genes related to the anthocyanin biosynthetic pathway and chlorophyll metabolic pathway.

## Results

### Total anthocyanin and total chlorophyll contents

**Fig. 1:**
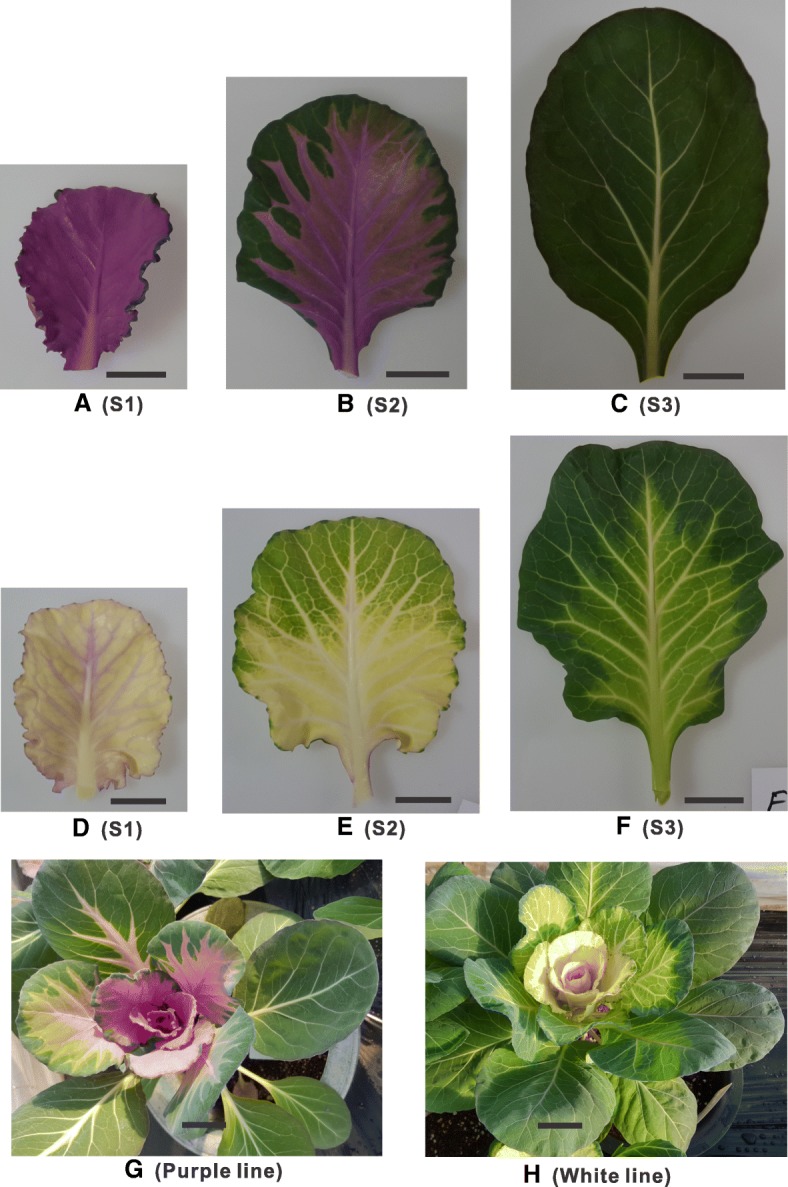
Pigment accumulation in leaves of purple and white ornamental cabbage lines at different stages of leaf development (S1-S3). S1, younger inner leaf; S2, mid-age leaf; S3, older leaf. Purple line (**a**-**c**): **a** younger purple leaf; **b** mid-age leaf (bicolor with purple at proximal end and green at distal end of the leaf); **c** older green leaf. White line (**d**-**f**): **d** younger white leaf; **e** mid-age leaf (bicolor with white center and green margin); **f** older light green leaf. **g** purple line (SCNU-OC-41-102) and (**h**), white line (SCNU-OC-30-28)

**Fig. 2:**
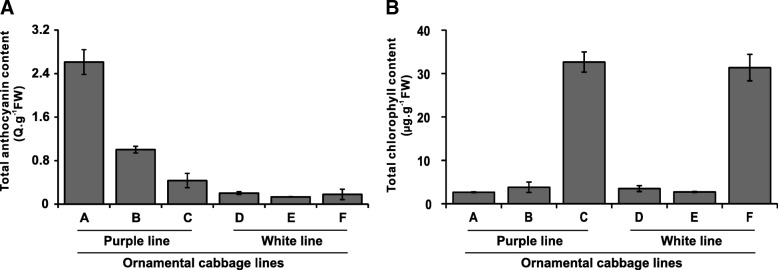
Total anthocyanin (**a**) and total chlorophyll (**b**) contents in purple and white ornamental cabbage at different stages of leaf development. Q, total anthocyanin content. Purple line: **a** younger purple leaf; **b** mid-age leaf (bicolor with purple at proximal end and green at distal end of the leaf); **c** older green leaf. White line: **d** younger white leaf; **e** mid-age leaf (bicolor with white center and green margin); **f** older light green leaf

We grew the cabbage advanced lines SCNU-OC-41-102 (purple ornamental cabbage) and SCNU-OC-30-28 (white ornamental cabbage) in a glasshouse and observed their development over time. Both ornamental cabbage lines underwent quick color changes in their leaves as development progressed. In the purple ornamental cabbage line, the younger and older leaves were purple and green, respectively, while mid-age leaves were bicolored with a purple center and a green margin (Fig. 1). Similarly, in the white ornamental cabbage line, the younger and older leaves were white and green, respectively, and the mid-age leaves were bicolored with a white center and a green margin (Fig. 1). We quantified the total anthocyanin and total chlorophyll contents in the leaves of both lines (Fig. 2). For anthocyanin, we found notable difference between the two lines. In the purple cabbage, the young inner leaves had the highest total anthocyanin content, around 2.5-fold higher than that in mid-age leaves, while the older leaves had the lowest anthocyanin content; in the white cabbage, there were no striking differences in anthocyanin content between developmental stages. Furthermore, the anthocyanin content in the younger leaves of the purple cabbage was over 10-fold higher than that in any developmental stage of the white cabbage (Fig. 2a). On the other hand, both the purple and white cabbage lines showed the highest chlorophyll content in older green leaves and extremely low chlorophyll in younger and mid-age leaves (Fig. 2b).

### Sequencing assembly and functional annotation

We obtained the transcriptomes of the six ornamental cabbage samples using Illumina sequencing technology. A total of 349.14 million paired-end raw reads (Table 1) were produced and deposited in the Sequence Reads Archive (SRA) of NCBI with the accession number SRP150110. Low-quality sequences, adapters, and ambiguous reads were removed and 339.75 million high-quality clean reads (Table 1) were obtained. These clean reads were assembled into 46,744 transcripts and 46,744 unigenes (Table 1).

**Table 1:** Overview of the ornamental cabbage transcriptome sequencing and assembly

Samples	Raw reads	Clean reads	Total mapped	Uniquely mapped	Reads, (+) strand	Reads, (−) strand	Splice reads
A	48,616,346	47,218,458 (97.1%)	41,796,549 (88.5%)	26,457,182 (56.0%)	13,228,359	13,228,823	8,422,139 (17.8%)
B	63,594,196	61,710,694 (97.0%)	54,668,668 (88.6%)	32,846,826 (53.2%)	16,420,999	16,425,827	10,265,355 (16.6%)
C	54,803,312	53,317,796 (97.3%)	45,812,394 (85.9%)	43,866,847 (82.3%)	21,956,864	21,909,983	15,395,923 (28.9%)
D	55,090,242	53,553,210 (97.2%)	47,042,034 (87.8%)	30,613,710 (57.2%)	15,297,123	15,316,587	9,825,925 (18.3%)
E	57,545,108	56,073,384 (97.4%)	47,979,679 (85.6%)	42,350,520 (75.5%)	21,187,624	21,162,896	15,050,563 (26.8%)
F	69,494,170	67,872,830 (97.7%)	57,395,716 (84.6%)	53,001,197 (78.1%)	26,566,170	26,435,027	18,136,612 (26.7%)
Total	349,143,374	339,746,372					
	Transcript	Gene					
Total number	46,744	46,744					

### Functional annotation and classification

**Fig. 3:**
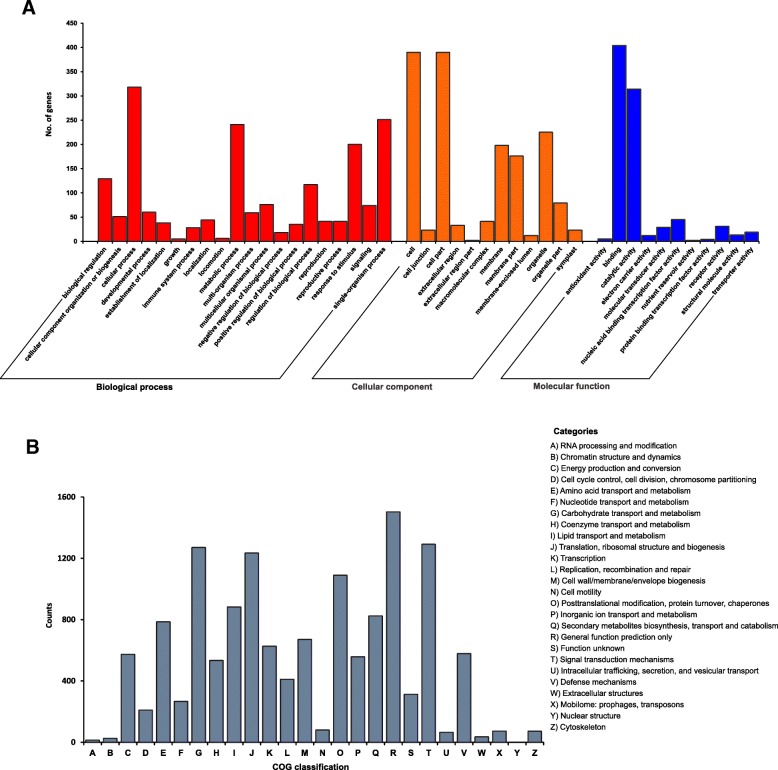
Functional categories of differentially expressed genes among different ornamental cabbage leaf samples. **a** GO classification; **b** COG classification of differentially expressed genes

We searched the unigenes against the SWISS-PROT database and in order to annotate the ornamental cabbage leaf transcriptomes. We performed Gene Ontology (GO) term enrichment (which classifies gene products into three main in main categories: molecular functions, biological processes, and subcellular compartments [28]) and Clusters of Orthologous Groups (COG) analysis to uncover the important biological functions of the products of the ornamental cabbage leaf transcriptome (Additional file 1: Table S1). The analysis of GO terms classified the differentially expressed unigenes into 43 important functional groups (Fig. 3a). Cellular process (GO:0009987), single-organism process (GO:0044699), metabolic process (GO:0008152) and response to stimulus (GO:0050896) were the most highly represented GO terms in the biological process category; cell (GO:0005623), cell part (GO:0044464), organelle (GO:0043226), membrane (GO:0016020) and membrane part (GO:0044425) represented the highest number of unigenes in the cellular component category; and binding (GO:0005488) and catalytic activity (GO:0003824) showed the highest numbers of unigenes for the molecular function category. The COG functional annotation categorized 12,475 unigenes into 26 COG classifications (Additional file 1: Table S1 and Fig. 3b). The five most prevalent COG categories represented in the ornamental cabbage transcriptomes were G (carbohydrate transport and metabolism), J (translation, ribosomal structure and biogenesis), O (post-translational modification, protein turnover and chaperones), R (general function prediction only) and T (signal transduction mechanisms) (Fig. 2b).

### Identification of differentially expressed genes

**Fig. 4:**
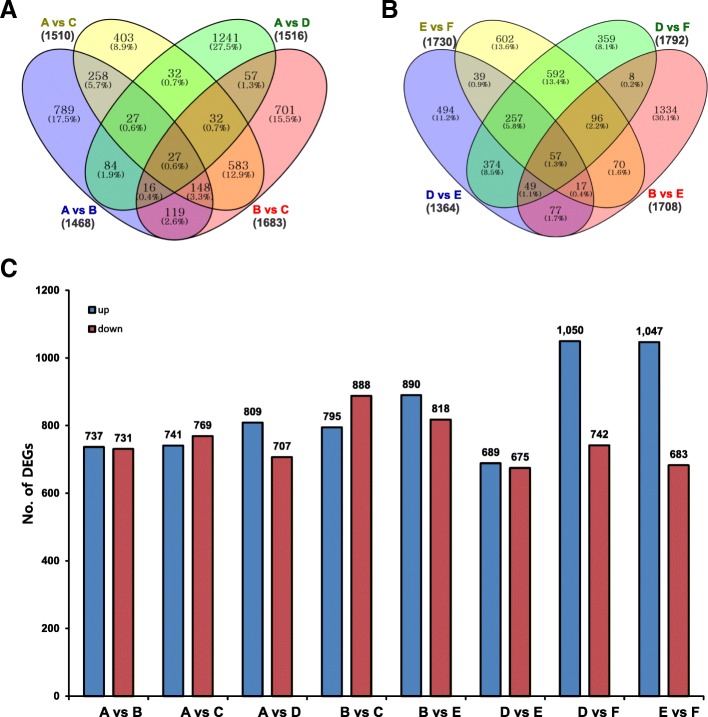
The differentially expressed genes identified in ornamental cabbage. Venn diagrams (**a** and **b**) showing the number of differentially expressed genes identified through pairwise comparisons. Number of up- and down-regulated genes in different pairwise comparisons (**c**). Venn diagram was generated using the freely available VENNY 2.1 online tool (http://bioinfogp.cnb.csic.es/tools/venny/)

**Fig. 5:**
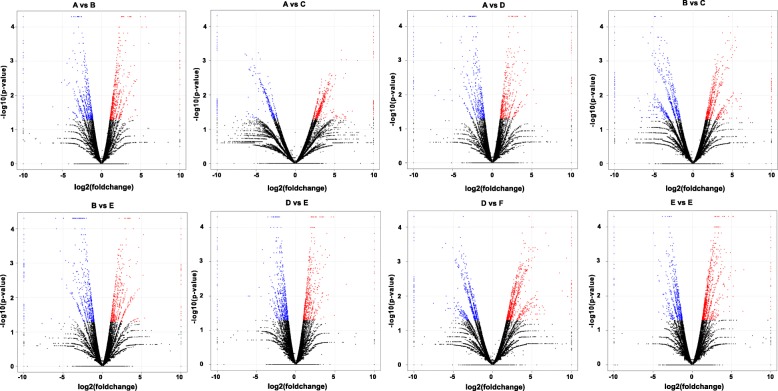
Volcano plots of differentially expressed genes (DEGs) in various pairwise comparisons during leaf developmental stages in ornamental cabbage. The *x* and *y* axes represent log_2_ fold change difference between the compared samples and statistical significance as the negative log of DEG *P*-values, respectively. The significantly up- and down-regulated genes are presented with red and blue dots, while non-significant genes as black dots

Using DEGseq, an R package, we identified the genes differentially expressed between purple and white ornamental cabbage lines at different leaf developmental stages (Additional file 2: Table S2) [29]. We identified a total of 12,771 differentially expressed genes (DEGs) by pairwise comparison (A vs B, A vs C, A vs D and B vs C, B vs E, D vs E, D vs F, E vs F) (Fig. 4a and Fig. b). The highest number of DEGs was found in D vs F (1792), with 1047 and 683 unigenes up- and down-regulated, respectively (Fig. 4c), among eight comparisons. In contrast, the lowest number of DEGs was observed in D vs E (1364), with 689 and 675 unigenes up- and down-regulated, respectively (Fig. 4c). Overall, 27 DEGs were common to the A vs B, A vs C, A vs D and B vs C comparisons, and 57 DEGs were common to the B vs E, D vs E, D vs F, E vs comparisons (Fig. 4a and b). Furthermore, volcano plots in Fig. 5 provide an overview of significantly differentially expressed genes. The red and blue dot in the volcano plots indicate significantly up- and down-regulated transcripts, respectively while black dots represent transcripts that are not differentially expressed.

### Expression pattern of anthocyanin biosynthetic genes

**Fig. 6:**
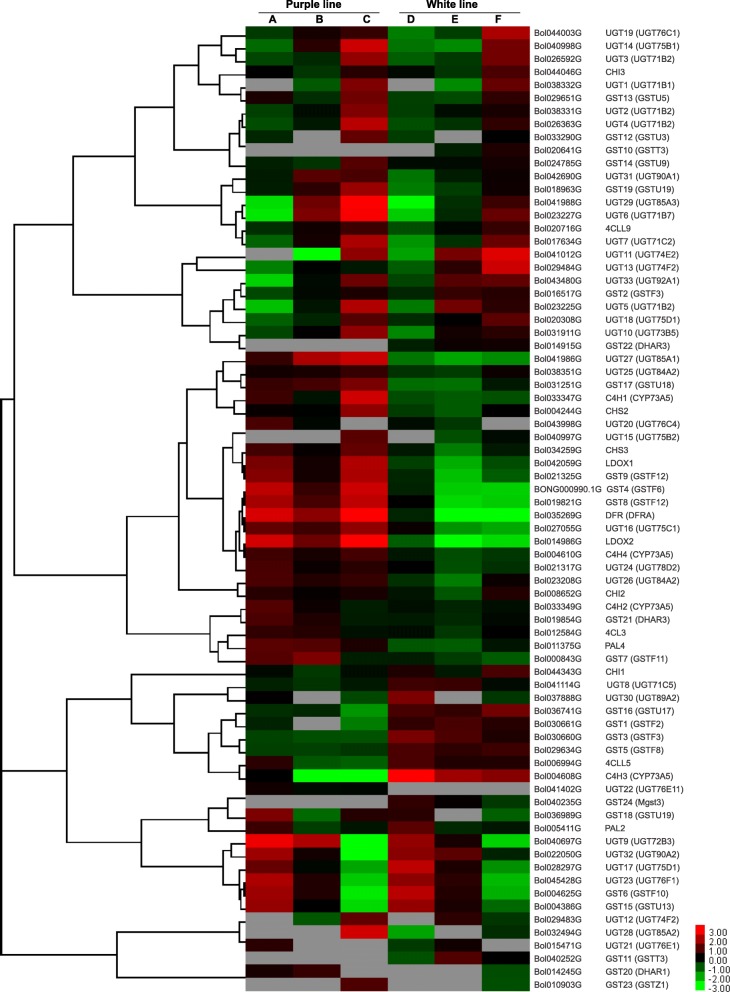
Heatmap representation of differentially expressed genes involved in anthocyanin biosynthesis in two ornamental cabbage lines. Fragments per kilobase of transcript per million mapped reads (FPKM) values are used to show the expression pattern. Purple line: **a** younger purple leaf; **b** mid-age leaf (bicolor with purple at proximal end and green at distal end); **c** older green leaf. White line: **d** younger white leaf; **e** mid-age leaf (bicolor with white center and green margin); **f** older light green leaf. The red and blue colors represent the maximum and the minimum values, respectively

The expression pattern of unigenes was determined by fragments per kilobase of exon per million mapped reads (FPKM) values. The purple and white ornamental cabbage lines underwent a rapid color change in their leaves across developmental stages (Fig. 1). In the present study, we analyzed the ornamental cabbage leaf transcriptomes for genes coding for enzymes related to the anthocyanin biosynthetic pathway. We identified a total of 278 unigenes involved in the anthocyanin pathway, of which 74 were differentially expressed (Table 2). Among these DEGs, *C4H2* (Bol033349G), *UGT9* (Bol040697G) and *GST21* (Bol019854G) showed higher expression in the inner leaves of purple ornamental cabbage in sample A than in any other samples, and very low expression in white ornamental cabbage samples (samples D, E, F) (Fig. 6). On the other hand, albeit the expression of *4CL3* (Bol012584G), *UGT24* (Bol021317G) was highest in purple inner leaf, nonetheless they had also higher expression levels in remaining leaf samples (B-D) of both the purple and white lines. Moreover, *UGT22* (Bol041402G) was only expressed in purple line, however a very little the expression differences was found among three stages (sample A-C; S1-S3). On the contrary, three unigenes (*C4H3*, Bol004608G; *GST2*2, Bol014915G; *GST24*, Bol040235G) were shown to be down-regulated in the purple line compared to the white line (Fig. 6).

**Table 2:** Candidate unigenes related to anthocyanin biosynthesis and chlorophyll metabolism in transcriptome of ornamental cabbage

Pathway	Gene	Description	No. of unigenes	No. of DEGs
Anthocyanin biosynthesis	*PAL*	Phenylalanine ammonia-lyase	9	2
	*C4H/CYP73A5*	Trans-cinnamate 4-monooxygenase	5	4
	*4CL*	4-coumarate--CoA ligase 2	27	3
	*CHS*	Chalcone synthase	8	2
	*CHI*	Chalcone-flavonone isomerase	5	3
	*F3H*	Naringenin,2-oxoglutarate 3-dioxygenase	7	0
	*F3’5’H*	Flavonoid 3′,5′-hydroxylase	1	0
	*DFR*	Dihydroflavonol 4-reductase	3	1
	*LDOX/ANS*	Leucoanthocyanidin dioxygenase/anthocyanidin synthase	7	2
	*UGT*	UDP-3-O-glucosyltransfersae	116	33
	*GST*	Glutathione S-transferase	90	24
Chlorophyll biosynthesis	*GluRS*	Glutamate-tRNA ligase	2	0
	*HEMA*	Glutamyl-tRNA reductase	6	2
	*GSA*	Glutamate-1-semialdehyde-2,1-aminomutase	2	0
	*HEMC*	Porphobilinogen deaminase	5	0
	*HEMD*	Uroporphyrinogen-III synthase	1	0
	*HEME*	Uroporphyrinogen decarboxylase	6	0
	*HEMF*	Coproporphyrinogen III oxidase	3	0
	*HEMG*	Oxygen-dependent protoporphyrinogen oxidase	2	0
	*CHLD*	Magnesium chelatase subunit D	1	0
	*CHLI*	Magnesium chelatase subunit I	3	2
	*CHLH*	Magnesium chelatase subunit H	1	0
	*CHLM*	Magnesium-protoporphyrin O-methyltransferase	2	0
	*CRD*	Magnesium-protoporphyrin IX monomethyl ester (oxidative) cyclase(oxidative) cyclase	3	1
	*POR*	Protochlorophyllide reductase	6	4
	*DVR*	Divinyl chlorophyllide-a 8-vinyl-reductase	1	0
	*ChlG*	Chlorophyll synthase	3	0
Chlorophyll cycle	*CAO*	Chlorophyllide a oxygenase	2	1
	*NYC1*	Chlorophyll(ide) b reductase	2	0
	*HCAR*	7-Hydroxymethyl chlorophyll a reductase	1	1
Chlorophyll degradation	*CLH*	Chlorophyllase	3	1
	*PaO*	Pheophorbide a oxygenase	1	0
	*RCCR*	Red chlorophyll catabolite reductase	1	1

### Identification of transcription factors (TFs)

We identified a total of 811 TFs belonging to 47 different TF families by aligning the DEGs to a plant TF database (PlantTFDB) with BLASTX (Table 3, Additional file 3: Table S3). Among these, thirteen (WRKY, C2H2, ERF, bHLH, B3, NAC, MYB, C3H, bZIP, CO-like, MYB_related, ARF and HD-ZIP) belonged to the major TF families, each of which comprises over 20 unigenes in ornamental cabbage. However, the largest number of unigenes (195) was identified for the WRKY TF family.

**Table 3:** Transcription factor (TF) families identified from the ornamental cabbage transcriptome

TF family	Number	TF family	Number
WRKY	195	BES1	6
C2H2	64	HSF	6
ERF	58	NF-YB	6
bHLH	51	CAMTA	4
B3	40	Dof	4
NAC	37	GRF	4
MYB	33	Nin-like	4
C3H	26	TALE	4
bZIP	25	WOX	4
CO-like	25	CPP	3
MYB_related	25	DBB	3
ARF	22	RAV	3
HD-ZIP	21	SBP	3
GATA	18	YABBY	2
LBD	17	E2F/DP	1
G2-like	15	FAR1	1
MIKC_MADS	13	GeBP	1
Trihelix	12	HRT-like	1
ARR-B	10	NF-X1	1
GRAS	9	NF-YA	1
TCP	9	NF-YC	1
ZF-HD	8	SRS	1
M-type_MADS	7	Whirly	1
AP2	6		

### Expression pattern of the regulatory genes of anthocyanin biosynthesis

**Fig. 7:**
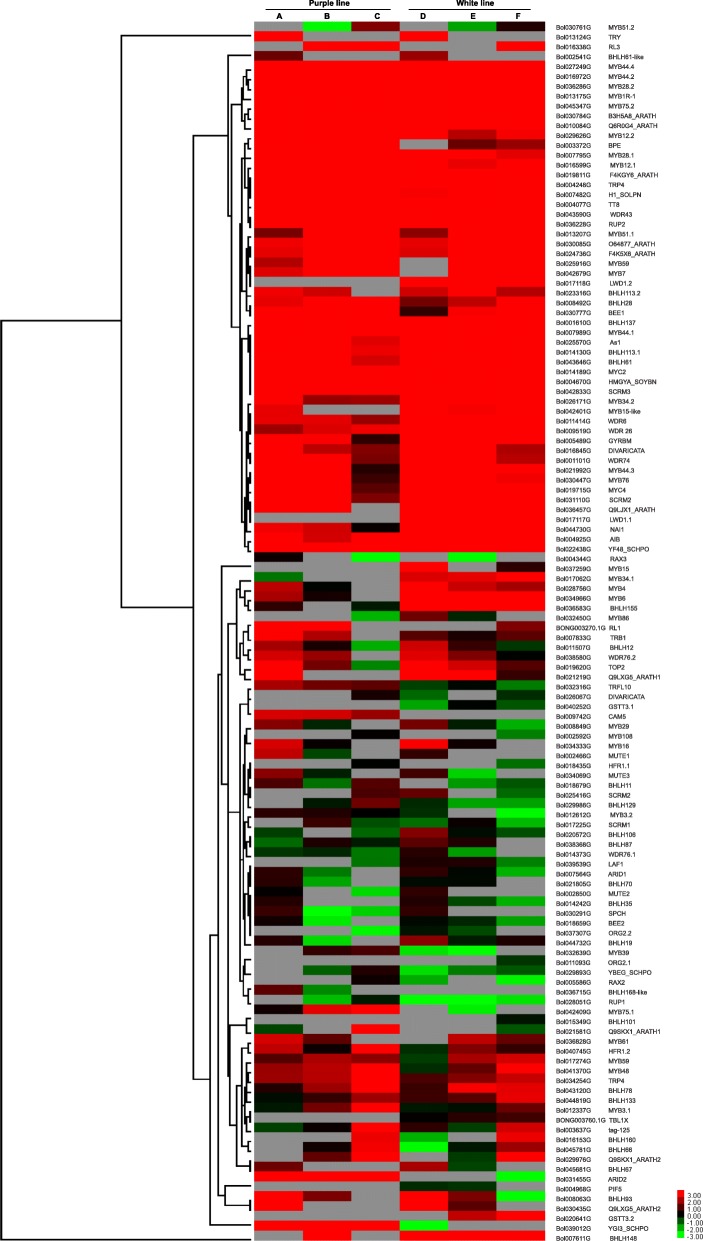
Heatmap representation of differentially expressed genes involved in regulation of anthocyanin biosynthesis in two ornamental cabbage lines. Fragments per kilobase of transcript per million mapped reads (FPKM) values are used to show the expression pattern. Purple line: **a** younger purple leaf; **b** mid-age bicolor leaf (bicolor with purple at proximal end and green at distal end); **c** older green leaf. White line: **d** younger white leaf; **e** mid-age leaf (bicolor with white center and green margin); **f** older light green leaf. The red and blue colors represent the maximum and the minimum values, respectively

The anthocyanin biosynthetic genes are known to be regulated by three different transcription factor (TF) families, the MYB, bHLH and WDR families. Therefore, we analyzed these TFs in this study. We detected a total of 537 unigenes for the aforementioned three categories of TF families and found that 124 of them were DEGs in our study: 58 MYBs (33 MYB family and 25 MYB-related), 51 bHLH and 15 WDR (Table 4). Among the 58 unigenes encoding MYB TFs, two (*RAX3,* Bol004344G; *TRB1*, Bol007833G) showed maximum transcript levels in the purple inner leaf (sample A, S1) compared to remaining five leaf samples (samples B-F) of both ornamental cabbage lines (Fig. 7). Two others, *MYB28.1* (Bol007795G) and *RL1* (BONG003270.1G) were highly expressed in the mid-aged purple/green bicolor leaf (sample B, S2), slightly decreased in the juvenile purple inner leaf (sample A, S1) and extremely low in the other four leaf samples (the older green leaf from the purple line, C (S3), and all three samples from the white cabbage line, D (S1), E (S2) and F (S3). However, the expression of two *MYB* genes (*TRFL10*, Bol032316G; *ARID2*, Bol031455G) was up-regulated in purple ornamental cabbage and down-regulated in white ornamental cabbage during all three leaf (S1-S3) developmental stages; and *MYB15*, Bol037259G, *MYB15-like* (Bol042401G), *MYB34.1* (Bol017062G), *MYB6* (Bol034966G) and *Q6R0G4_ARATH* (Bol010084G) showed the opposite expression pattern, being down-regulated in purple cabbage and up-regulated in white cabbage at all stages (Fig. 7). In case of *bHLH*s, out of 51 DEGs, only two *bHLH* genes (*MUTE1*, Bol002466G; *BHLH168-like,* Bol036715G) were up-regulated in purple inner leaf (sample A, S1) and down-regulated in the rest of the leaf samples (B, C, D, E and F) (Fig. 7). In addition, two *bHLH* genes (*BPE*, Bol003372G; *TT8*, Bol004077G; *CAM5*, Bol009742G) were up-regulated in purple ornamental cabbage but down-regulated in white ornamental cabbage throughout all three leaf developmental stages (S1-S3), and two unigenes (*BHLH155,* Bol036583G and *NAI1,* Bol044730G) showed the opposite trend of being down-regulated in purple cabbage but up-regulated in white cabbage (samples D-F, S1–3). Among the *WDR* genes, only *WDR43* (Bol043590G) showed differential expression correlating with ornamental cabbage line and stage, with highest expression in purple inner leaf (sample A, S1) and to a lesser degree in purple/green bicolor leaf (sample B, S2), lower expression in older green leaf from the purple cabbage line (sample C, S3) and white inner leaf (sample D, S1), and extremely low expression in white/green bicolor leaf (sample E, S2) and older green leaf from the white cabbage line (sample F, S3) (Fig. 7).

**Table 4:** Differentially expressed MBW-complex-forming MYB, bHLH and WD repeat transcription factor families in ornamental cabbage

Regulatory gene	Description	No. of unigenes	No. of DEGs
*MYB*	MYB-like transcription factor (TF)	230	33
*bHLH*	Basic helix-loop-helix TF	213	25
*WDR*	WD repeat-containing protein	94	15

### Expression patterns of genes related to chlorophyll metabolism

**Fig. 8:**
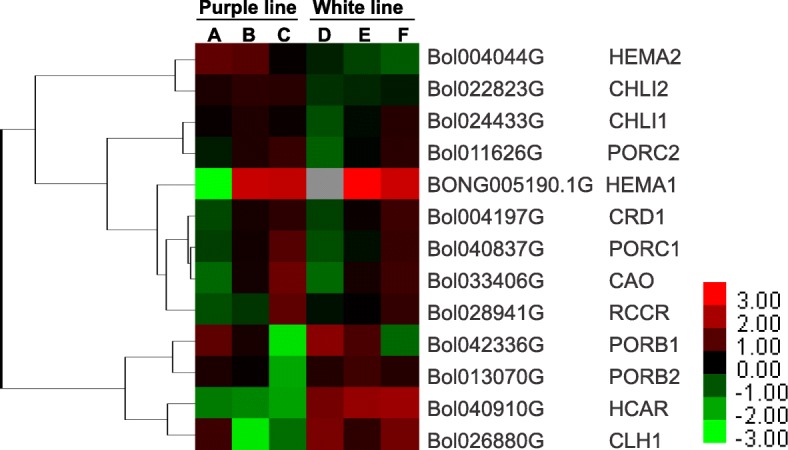
Heatmap representation of differentially expressed genes involved in chlorophyll biosynthesis in two ornamental cabbage lines. Fragments per kilobase of transcript per million mapped reads (FPKM) values are used to show the expression pattern. Purple line: **a** younger purple leaf; **b** mid-age leaf (bicolor with purple at proximal end and green at distal end); **c** older green leaf. White line: **d** younger white leaf; **e** mid-age leaf (bicolor with white center and green margin); **f** older light green leaf. The red and blue colors represent the maximum and the minimum values, respectively

In both the purple and white ornamental cabbage lines, the younger inner leaves (S1) were purple or white, respectively, but the mid-age leaves (S2) started to become bicolored as chlorophyll accumulated, and the older leaves of both lines were fully green. Therefore, we also analyzed genes related to chlorophyll metabolism (S3). We found a total of 57 unigenes related to chlorophyll metabolism, of which 13 were DEGs in ornamental cabbage (Table 2). Among these DEGs were nine genes involved in chlorophyll biosynthesis, of which five (*HEMA1*, BONG005190.1G; *CRD1*, Bol004197G; *PORC1*, Bol040837G; *PORC2*, Bol011626G) showed higher expression in mid-age and older leaves (sample B, S2; sample C, S3; sample E, S2; sample F, S3) of both the purple and white ornamental cabbage lines, but extremely low expression in purple (sample A, S1) and white inner, juvenile leaves (sample D, S1) (Fig. 8). The differentially expressed gene *CAO* (Bol033406G) related to the chlorophyll cycle showed a similar expression pattern (Fig. 8). In addition, *HCAR* (Bol040910G) involved in chlorophyll cycle was shown to be down-regulated in purple line (sample A-C, S1-S3) compared to white line. Meanwhile, a differentially expressed gene related to chlorophyll degradation, *CLH1* (Bol026880G), showed contrasting expression patterns in the purple and white ornamental cabbage lines. In purple ornamental cabbage, its expression was high in the juvenile inner leaves (sample A, S1) but decreased in mid-age (sample B, S2) and older leaves (sample C, S3). On the other hand, in white ornamental cabbage, expression of *CLH1* was very high in juvenile inner (sample D, S1) and outer older leaves (sample F, S3), and low in mid-age leaves (sample E, S2) (Fig. 8).

### KEGG enrichment analysis of the DEGs

**Fig. 9:**
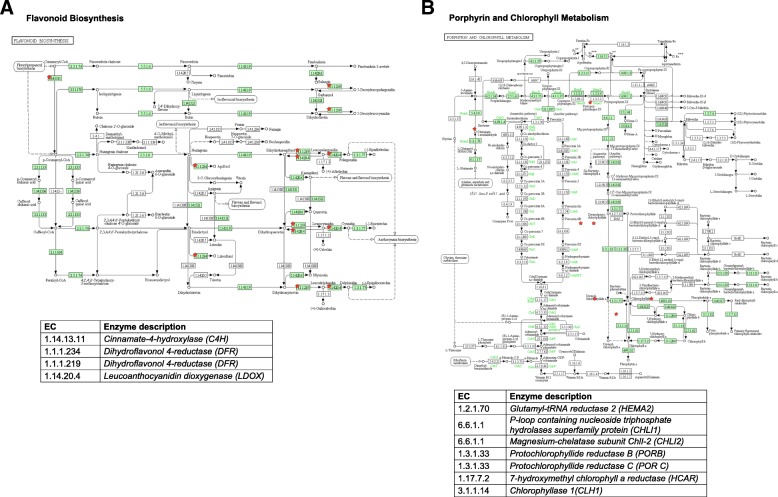
KEGG pathway showing flavonoid biosynthesis (**a**) and chlorophyll metabolism (**b**) in ornamental cabbage. The catalytic enzymes involved in these pathways are indicated by red asterisks. The description of enzyme accessions are provided below each pathway

We performed KEGG pathway enrichment of the DEGs using the WebGestalt online tool freely available at http://www.webgestalt.org (Table 5). The results showed that the important enriched pathways were biosynthesis of secondary metabolites, carbon metabolism, cysteine and methionine metabolism, glyoxylate and dicarboxylate metabolism, glycine, serine and threonine metabolism, and photosynthesis. Among these, the secondary metabolite biosynthesis pathway was the most notable enriched pathway, including a total 264 unigenes. There were nine and eight unigenes related to the anthocyanin biosynthesis pathway (*PAL2*, Bol005411G; *PAL4*, Bol011375G; *C4H2*, Bol033349G; *4CL3*, Bol012584G; *DFR*, Bol035269G; *LDOX*, Bol042059G; *UGT74B1*, Bol005786G; *UGT76C1*, Bol044003G; *UGT78D2*, Bol021317G) and to chlorophyll metabolism (chlorophyll biosynthesis: *HEMA2*, Bol004044G; *CHLI1*, Bol024433G; *CHLI2*, Bol022823G; *PORB1*, Bol042336G; *POR C2*, Bol011626G; chlorophyll cycle: *HCAR*, Bol040910G; chlorophyll degradation: *CLH1*, Bol026880G), respectively (Fig. 9 and Additional file 4: Table S4).

**Table 5:** KEGG pathways enriched in the differentially expressed genes

KEGG pathway	No. of genes	FDR^a^ threshold
Biosynthesis of secondary metabolites	264	2.82E-07
Glucosinolate biosynthesis	13	1.25E-04
Cysteine and methionine metabolism	40	2.53E-04
Carotenoid biosynthesis	15	1.16E-03
Glyoxylate and dicarboxylate metabolism	28	1.16E-03
Carbon metabolism	70	5.61E-03
Glycine, serine and threonine metabolism	25	9.88E-03
Tyrosine metabolism	16	1.54E-02
Photosynthesis	25	2.27E-02
Linoleic acid metabolism	6	2.27E-02

^a^FRD, false discovery rate

### Validation of differential gene expression by qRT-PCR

**Fig. 10:**
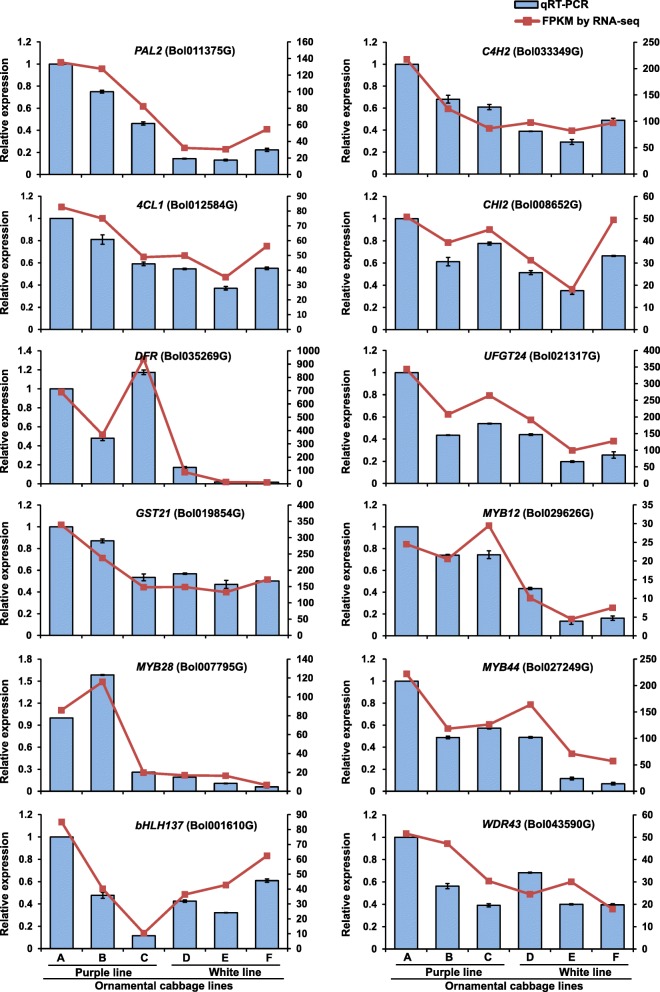
Relative expression of twelve unigenes related to anthocyanin biosynthesis during different leaf developmental stages in two ornamental cabbage lines. Error bar represents ±SE of the means of triplicates. Purple line: **a** younger purple leaf; **b** mid-age leaf (bicolor with purple at proximal end and green at distal end); **c** older green leaf. White line: **d** younger white leaf; **e** mid-age leaf (bicolor with white center and green margin); **f** older light green leaf. The RNA-seq expression profiles (FPKM) were presented as line graph about the bars

**Fig. 11:**
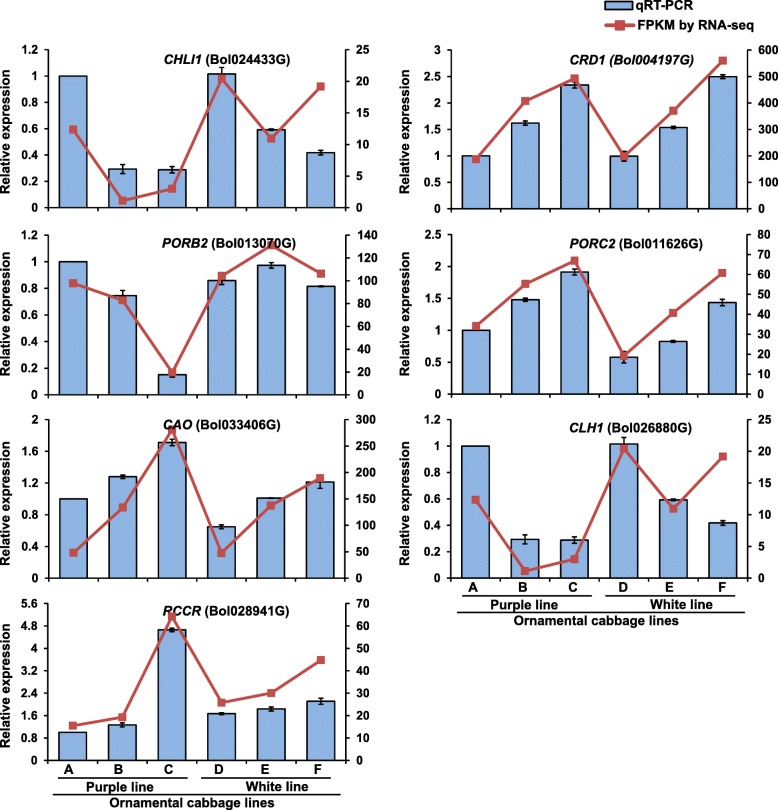
Relative expression of seven unigenes related to chlorophyll metabolism during different leaf developmental stages in two ornamental cabbage lines. Error bar represents ±SE of the means of triplicates. Purple line: **a** younger purple leaf; **b** mid-age leaf (bicolor with purple at proximal end and green at distal end); **c** older green leaf. White line: **d** younger white leaf; **e** mid-age leaf (bicolor with white center and green margin); **f** older light green leaf. The RNA-seq expression profiles (FPKM) were presented as line graph about the bars

Next, we further validated the expression patterns of DEGs (obtained via RNA-seq) by quantitative real time PCR (qRT-PCR). To do this, we randomly selected 19 differentially expressed genes and analyzed them using gene-specific primers (Additional file 5: Table S5). The qRT-PCR analysis confirmed that the selected genes were differentially expressed in purple and white lines (Figs. 10 and 11). Further, the expression of the most the structural and regulatory genes of anthocyanin biosynthesis were maximum in younger inner leaves (sample A, S1) followed by mid-age leaves (sample B, S2) of purple ornamental cabbage line compared to white line (Fig. 10). The result indicated the highest level of anthocynins younger inner leaves of purple line. In addition, two chlorophyll biosynthetic genes (*CRD1*, Bol004197G and *PORC2*, Bol011626G) showed their maximum expression in older leaves (sample C, S3 and sample F, S3) suggested higher levels of chlorophyll biosynthesis in older green leaves of both purple and green lines (Fig. 11). Moreover, *CLH1* (Bol026880G) code for a chlorophyll degrading enzyme *chlorophyllase* was highly expressed in younger inner leaves (sample A, S1 and sample D, S1) indicating higher rates of chlorophyll degradation in purple and white inner leaves of purple and white lines, respectively (Fig. 11).

### Expression patterns of candidate unigenes in bicolor leaves

**Fig. 12:**
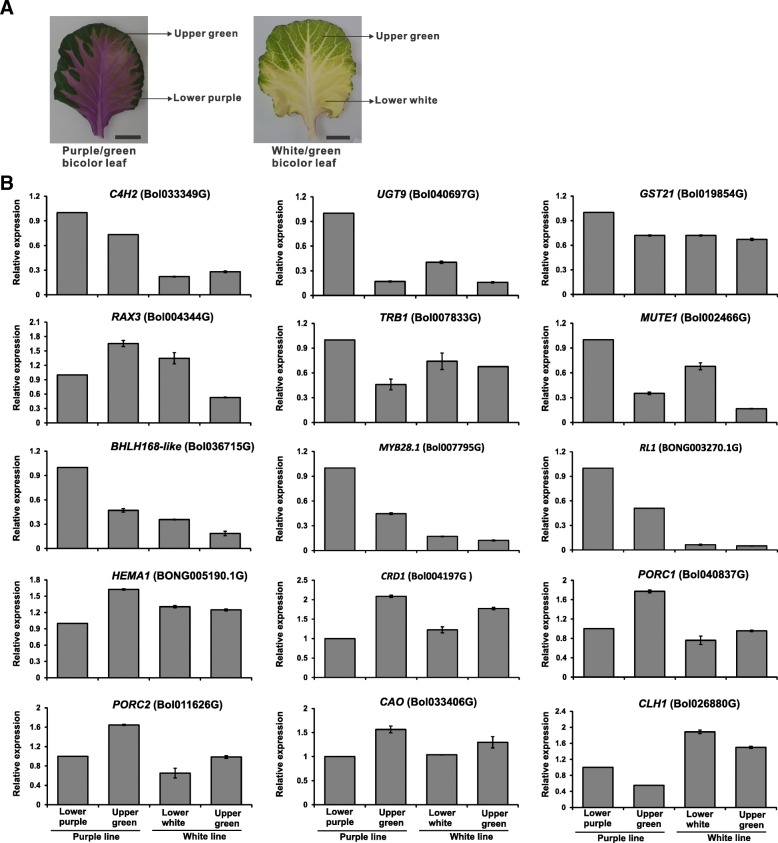
Relative expression of candidate unigenes related to anthocyanin biosynthesis and chlorophyll metabolism in mid-age bicolor (purple/green and white/green) leaves of two ornamental cabbage lines. **a** mid-age bicolor leaves; **b** qRT-PCR expression. Error bar represents ±SE of the means of triplicates

The bicolor leaves of ornamental cabbage lines were separated as lower purple and upper green areas for purple line while lower white and upper green areas for white line. The expression pattern of candidate unigenes was analyzed by qRT-PCR (Fig. 12). The expression level of candidate structural genes for anthocyanin biosynthesis, including *C4H2* (Bol033349G), *UGT9* (Bol040697G) and *GST21* (Bol019854G) was relatively higher in lower purple area than in upper green area of purple/green, and both lower white and upper green areas of white/green bicolor leaves. Unlike structural genes, the expression level of candidate regulatory genes (*TRB1*, Bol007833G; *MUTE1*, Bol002466G; *bHLH168-like,* Bol036715G) was also higher in lower purple area of purple/green bicolor leaf except *RAX3 (*Bol004344G). Moreover, two MYB genes (*MYB28.1*, Bol007795G; *RL1*, BONG003270.1G) were up-regulated in purple area of purple/green bicolor leaf suggests that *MYB28.1* and *RL1* might be involved in purple pigmentation of purple/green leaf. On the other hand, candidate ungenes related to chlorophyll biosynthesis (*HEMA1*, BONG005190.1G; *CRD1*, Bol004197G; *PORC1*, Bol040837G; *PORC2*, Bol011626G) and chlorophyll cycle (CAO, Bol033406G) showed relatively higher expression levels in the upper green area of purple/green than both lower white and upper green areas of white/green bicolor leaves. Moreover, *CLH1* (Bol026880G) gene related to chlorophyll degradation showed transcript abundance in both lower white and upper green areas of white/green bicolor indicates a higher rate of chlorophyll degradation in these areas.

## Discussion

In recent years, ornamental cabbage has become popular as a decorative plant for its fascinating foliage color and ability to withstand cooler winters. The advances in next-generation sequencing technologies have made it possible to perform large-scale high-throughput genome and transcriptome analysis. Transcriptome sequencing technology is a powerful tool for understanding the molecular basis of plant responses under various conditions and has been successfully applied in several plants [30, 31]. Several studies have been carried out in *Brassica oleracea* subspecies including cabbage, cauliflower and broccoli [32–34]. Moreover, some research has identified anthocyanin-related genes in lily, pak choi, tree peony, sweet cherry and kiwifruit [27, 35–38]. Nevertheless, up until now there has been no report of the transcriptome analysis of ornamental cabbage and specifically the purple leaf pigmentation produced by anthocyanins. In this study, we performed transcriptomic analysis to investigate the variations in transcription patterns in relation to leaf pigmentation during development in two ornamental cabbage lines (purple and white) by RNA-seq. Our results not only identified candidate biosynthetic and regulatory genes involved in anthocyanin biosynthesis but also provided insight into bicolor leaf development in purple ornamental cabbage.

We obtained 349.14 million paired-end raw reads (339.75 million clean reads) and assembled them into 46,744 unigenes (Table 1), a process that provides a valuable resource for large-scale investigation of gene function and was enlightening in regard to anthocyanin biosynthesis as well as chlorophyll metabolism during the leaf developmental process in ornamental cabbage. In particular, we identified 74 and 57 differentially expressed putative candidate unigenes related to the anthocyanin biosynthetic pathway and chlorophyll metabolism, respectively (Table 2). Among them, in many cases, more than one unigene was annotated for a single enzyme. The exceptions were *DFR*, *CRD*, *CAO*, *HCAR*, *CLH* and *RCCR*, suggesting that these unigenes might be denoted as different members of a particular gene family or different fragments of a specific transcript, or both [39–41].

In the case of anthocyanin biosynthetic genes, we found three unigenes, *C4H2*, *UGT9* and *GST21*, that were up-regulated in purple inner leaves (sample A, S1) and to a lesser degree in purple/green bicolor mid-age leaves (sample B, S2) but down-regulated in older green leaves (sample C, S3) of purple ornamental cabbage (Fig. 6). All three were much more weakly expressed in the white ornamental cabbage line, being slightly expressed in white inner leaves (sample D, S1) but down-regulated at the other two developmental stages (samples E-F; S2-S3) in white ornamental cabbage (Fig. 6). In the younger white inner leaves (sample D, S1) of white ornamental cabbage, a very small amount of anthocyanin pigmentation was observed (Fig. 1d). This may be due to the slight expression of the aforementioned anthocyanin biosynthetic genes in white inner leaves of this cabbage line. This result suggests that the up-regulation of the structural genes of the anthocyanin pathway might responsible for the purple pigmentation in the leaves of purple ornamental cabbage. Similar results were found by Xu et al. [40] for purple pigmentation in the ovary of Asiatic hybrid lilies. Xu et al. [27] reported that the formation of purple bases in the bicolor tepals of the lily cultivar ‘Tiny Padhye’ was due the up-regulation of structural genes of the anthocyanin pathway.

Previous studies have revealed that the structural genes of the anthocyanin pathway are controlled by the MBW complex [7, 15, 21]. The up-regulation of two candidate *MYB* genes, *RAX3* (Bol004344G) and *TRB1* (Bol007833G) in purple inner leaf (Fig. 7) reveals that these unigenes might be important to the purple pigmentation of purple cabbage inner leaf. Moreover, the unigenes *MYB28.1* (Bol007795G) and *RL1* (BONG003270.1G) showed up-regulation in younger purple inner leaf and mid-age purple/green bicolor leaf (purple center with green margin) (Fig. 7 and Additional file 6: Figure S1) implies that these unigene might play an important role in the purple pigmentation of inner leaf in ornamental cabbage. However, in *Arabidopsis* there are four MYB TFs known to involved in the regulation of anthocyanin biosynthesis (AtMYB75/PAP1, AtMYB90/PAP2, AtMYB113 and AtMYB114) [42]. Jin et al. [3] reported three orthologues of these *Arabidopsis MYB* genes in ornamental cabbage, namely *BoPAP1, BoPAP2* and *BoMYB114*, of which *BoPAP2* was the candidate regulatory gene for anthocyanin biosynthesis in ornamental cabbage. Our results indicate that only *MYB75* was differentially expressed in ornamental cabbage, but it showed maximum expression in older green leaf. In *Arabidopsis*, three MYB TFs, AtMYB11, AtMYB12 and AtMYB111 TFs, are known to be involved in the activation of early structural genes of the anthocyanin pathway [42]. In this study, we found only one TF unigene *BoMYB12.2* (Bol029626G), an orthologue of *AtMYB12* showed higher expression in purple as compared to white ornamental cabbage. Zhang et al. [35] also previously reported only MYB11 was up-regulated in pak choi when compared with *Arabidopsis* (AtMYB11, AtMYB12 and AtMYB111). In *Arbidopsis*, AtMYB3 is known to repress phenylpropanoid biosynthesis [43]. The unigene *MYB3.1* (Bol012337G), an orthologue of *AtMYB3* showed its maximum transcript abundance in older green leaf (sample C, S3) of purple line (Fig. 7 and Additional file 6: Figure S1). This suggests that *MYB3.1* might inhibit anthocyanin biosynthesis in the older green leaf of purple ornamental cabbage. In addition, the unigene Bol007204 encodes a MYBL2 TF, a negative regulator of flavonoid biosynthesis [43] that showed lowest expression in purple inner leaf (sample A, S1) of purple ornamental cabbage and a pattern of increasing over time during the remaining developmental stages of both ornamental cabbage lines, peaking in the white/green bicolor leaf (sample E, S2) (Additional file 6: Figure S1). Two *bHLH* genes (Bol002466G and Bol036715G) were up-regulated in purple inner leaves but were barely detectable or undetectable in the remaining samples of both ornamental cabbage lines (Fig. 7). Furthermore, the expression of *TT8* (Bol004077G) was up-regulated in purple and down-regulated in white ornamental cabbage across the developmental stages. Thus suggesting their possible involvement of these *bHLH* genes in the regulation of the structural genes of anthocyanin biosynthesis in purple ornamental cabbage. These data will help us to elucidate the molecular mechanisms of anthocyanin biosynthesis in the inner leaf of ornamental cabbage. Notably, it has been reported that TT8 was potentially involved in the regulation of anthocyanin biosynthetic genes in pak choi [35].

Out of 57 unigenes related to chlorophyll metabolism, 13 were differentially expressed in ornamental cabbage (Table 2). The unigenes related to chlorophyll biosynthesis (*HEMA1*, BONG005190.1G; *CRD1*, Bol004197G; *PORC1*, Bol040837G; *PORC2*, Bol011626G) and the chlorophyll cycle (*CAO*, Bol033406G) were up-regulated in mid-age (S2) and older (S3) leaves in both the ornamental cabbage lines (Fig. 8), similar to what has been reported by Ren et al. [44] for Chinese narcissus. On the other hand, *CLH1* (Bol026880G), a gene involved in chlorophyll degradation, showed an opposite expression pattern except in the older leaf (sample F, S3) of white ornamental cabbage. The older green leaf (sample F, S3) of white ornamental cabbage was whitish green, with less green pigment than the older leaf (sample C, S3) of purple ornamental cabbage (Fig. 1f). This might be the reason for higher expression of *CLH1* in older leaf (sample F, S3) of white ornamental cabbage. These results suggest that up-regulation of chlorophyll-degradation-related genes and down-regulation of chlorophyll-biosynthesis-related genes cause degradation of chlorophyll in younger inner (S1) and bicolor mid-age (S2) leaves of both the ornamental cabbage lines. Our results are in agreement with observations of Xu et al. [27] in lily.

We detected numerous differentially expressed unigenes for some TFs, including WRKY, C2H2, ERF, bHLH, B3, NAC, MYB, C3H, bZIP, CO-like and MYB-related, indicating that these TFs might be involved in the purple younger inner and mid-age bicolor leaf pigmentation in ornamental cabbage. Verweij et al. [45] reported that WRKY44 (AtTTG2) regulates the color patterns of petals in *Petunia* via acidification of the cell vacuole. It has been reported that WRKY and NAC TFs regulate chlorophyll degradation [27, 46].

Furthermore, the expression level of candidate unigenes was further analyzed in bicolor (purple/green and white/green) leaves. The expression pattern of candidate genes related to anthocyanin biosynthesis and chlorophyll metabolism revealed that these genes might involve in bicolor leaf development in ornamental cabbage (Fig. 12). Zhao et al. [39], upon analyzing the transcriptomes of Chinese traditional flower peonies in which the outer and inner petals were red and yellow, respectively, also found that the expression of anthocyanin biosynthetic genes was higher in outer petals and lower in inner petals. Zhang et al. [35] also reported that the structural genes had higher expression in a pak choi cultivar with purple leaves than in one with green leaves.

## Conclusion

We carried out transcriptome analysis by RNA-seq to understand the molecular mechanism underlying the development of the purple inner leaf and the bicolor leaf with purple center and green margin in purple ornamental cabbage, as well as the white inner leaf and bicolor leaf with white center and green margin in white ornamental cabbage. Our results suggest that the purple inner leaves of purple ornamental cabbage are due to the combination of high level of anthocyanin biosynthesis, high level of chlorophyll degradation and extremely low chlorophyll biosynthesis at that developmental stage compared to other stages. The purple/green bicoloration is due to a moderate level of anthocyanin biosynthesis, high chlorophyll degradation and a very low level of chlorophyll biosynthesis. On the other hand, the white inner leaves of white ornamental cabbage are due to the combination of extremely low level or absence of anthocyanin biosynthesis, high level of chlorophyll degradation and low of chlorophyll biosynthesis. The bicolor leaves with a white center and green margin is due to high level of chlorophyll degradation, low level of chlorophyll biosynthesis and almost background level or absence of anthocyanin biosynthesis. These results provide insight into the molecular mechanisms of inner purple or white and outer bicolor leaf pigmentation, which might be helpful for further understanding of leaf color development and for the breeding of new colorful ornamental cabbage cultivars.

## Methods

### Experimental plant materials

The ornamental cabbage advanced lines SCNU-OC-41-102 (purple inner leaf) and SCNU-OC-30-28 (white inner leaf) were used in this study. The plants were grown in a glasshouse at the Department of Horticulture, Sunchon National University, Suncheon, Republic of Korea. Leaf samples were collected from leaves of both lines (60 days after sowing) at three distinct developmental stages (Fig. 1): stage 1, younger inner, i.e. 18th leaf (A, purple; D, white); stage 2, purple/green mid-age leaf, i.e. 14th leaf (B, purple center with green leaf margin; E, white center with green leaf margin); and stage 2, fully expanded older 10th leaf (C, dark green; F, light green). Leaf number was counted from the outer, oldest leaves to the inner, youngest leaves. Thereafter, the collected samples were frozen in liquid nitrogen and stored at − 80 °C until use.

### Quantification of total anthocyanins and total chlorophyll contents

Total anthocyanins were quantified from the leaves of ornamental cabbage by extraction with methanol (CH_3_OH) containing 1% HCl. The leaves were pulverized in liquid nitrogen, moved into an eppendorf tube containing methanol/1% HCl, and left overnight at room temperature. Thereafter, the extract was centrifuged at 14,000 rpm for 10 min and the absorbance was determined spectrophotometrically (wavelengths 530 and 657 nm). The anthocyanin concentrations were stated as Q = (A_530_–0.25 × A_657_) × FW^− 1^, where Q = total anthocyanins; A_530_ = absorption at 530 nm; A_657_ = absorption at 657 nm; FW = fresh weight of leaves (g) [47]. Total chlorophyll content was determined using 200 mg ornamental cabbage leaves (grounded into powder in liquid nitrogen) by methanol (10 ml 100% pure solvent) extraction [48]. Then, the suspension was centrifuged at 14,000 rpm for 5 min. The optical density of the supernatant was measured with the spectrophotometer at 652.4 and 665.2 nm and total chlorophyll content was measured using the formula (total chlorophyll content = 1.44A_665.2–_24.93A_652.4_, where, A_665.2_ = absorption at 665.2 nm and A_652.4_ = absorption at 652.4 nm) previously reported by Lichtenthaler [48].

### Total RNA extraction, library construction, sequencing, and transcriptome assembly

The leaf samples of ornamental cabbage for each developmental stage were ground into a fine powder in liquid nitrogen. Then, total RNA was extracted from 100 mg powder using RNeasy mini kit (Qiagen, USA) following the manufacturer’s instructions. The quantity and integrity were checked with a NanoDrop spectrophotometer (NanoDrop Technologies, Wilmington, Delaware, USA) and Agilent 2100 BioAnalyzer (Agilent Technologies, Palo Alto, CA, USA). Total RNA samples with an RNA integrity number (RIN) of higher than 7 were used to prepare RNA-seq libraries. A total of six RNA-seq libraries were constructed using TruSeq RNA Library Prep Kit (Illumina Inc.) by Theragen Bio Institute (Suwon, South Korea), and RNA sequencing was carried out using an Illumina HiSeq 2000 high throughput sequencing system (Illumina Inc.). Thereafter, the RNA sequencing data were analyzed according to the method described Trapnell et al. [49].

### Functional classification

The assembled unigenes were searched against the Swiss-Prot (http://www.expasy.ch/sprot/), Gene Ontology (GO) [50], Eukaryotic Ortholog Groups (KOG) [51] and Kyoto Encyclopedia of Genes and Genomes (KEGG; KEGG(http://www.genome.jp/kegg/) databases.

### Quantification of gene expression levels and differentially expressed genes

The clean reads were mapped to the reference genome known as *Brassica oleracea* Genome Database (Bolbase, http://www.ocri-genomics.org/bolbase/) using TopHat v.2.1.1 (http://ccb.jhu.edu/). The gene expression levels and differential expression were determined using the Cufflinks program v.2.0.1 (http://cufflinks.cbcb.umd.edu/) as previously reported by Trapnell et al. [49]. The gene expression levels were normalized by the number of fragments per kilobase of exon per million mapped reads (FPKM). The FPKM value for each gene was determined based on the length of the gene and reads count mapped to this gene. The differentially expressed genes (DEGs) were identified by using DEGseq, an R based program for detecting DEGs from RNA-seq data [29]. The standard value for selecting DEG was adjusted at *p* < 0.005 and *q* < 0.05. Furthermore, the differentially expressed genes were subjected to pathway enrichment and network analysis using WebGestalt [52].

### cDNA synthesis and qPCR validation

A total of 1 μg high quality total RNA was converted into cDNA using SuperScript® III following the manufacturer’s instruction (Invitrogen, Gaithersburg, MD). Then, 30 unigenes related to anthocyanin biosynthesis and 20 unigenes related to chlorophyll metabolism were selected for further validation. The validation of selected genes was carried out by qRT-PCR with ‘LightCycler®96’ (Roche, Mannheim, Germany). A total of 50 ng cDNA was used for qRTPCR reaction using gene-specific primers (Additional file 5: Table S5) with ‘2x SyGreen Mix Lo-ROX (qPCRBIO)’ (PCR Biosystems, London, UK). The reaction conditions were set at 95 °C for 5 min, 50 cycles of 95 °C for 10 s, 60 °C for 10 s, 72 °C (15 s). At the end of PCR cycles, the Cq values were analyzed with LightCycler® 96 software (Roche, Germany). The relative expression was determined using the comparative 2^−ΔΔCt^ method [53] with the *actin* gene considered as internal control.

### Identification of transcription factors (TFs)

TFs were identified from the differentially expressed genes (DEGs) using plant specific PlantTFDB (http://planttfdb.cbi.pku.edu.cn/) with BLASTX (E-value cut-off of ≤10^− 5^).

## Additional files

Additional file 1: **Table S1.** Functional annotation and classification of unigenes. (XLSX 5689 kb)

Additional file 2: **Table S2.** Differentially expressed genes (DEGs) found in pairwise comparisons in leaf samples of two ornamental cabbage lines. (XLSX 1710 kb)

Additional file 3: **Table S3.** Transcription factor (TF) families identified in two ornamental cabbage lines. (XLSX 98 kb)

Additional file 4: **Table S4.** Unigenes related to secondary metabolites biosynthesis. (XLSX 26 kb)

Additional file 5: **Table S5.** List of gene-specific primers used for qRT-PCR validation. (XLSX 10 kb)

Additional file 6: **Figure S1.** Expression pattern of *MYB28.1, RL1* and *MYBL2* using FPKM (fragment per kilobase of transcript per million mapped reads) values of ornamental cabbage leaf transcriptome. A, younger purple leaf; B, mid-age leaf (bicolor with purple at proximal end and green at distal end); C, older green leaf; D, younger white leaf; E, bicolor mid-age leaf (bicolor with white center and green margin); F, older light green leaf. (DOCX 144 kb)

## Abbreviations

4CL: 4-coumarate--CoA ligase 2
*ANR*: *Anthocyanidin reductase*
bHLH: Basic helix-loop-helix
*C4H*: *Trans-cinnamate 4-monooxygenase*
*CAO*: *Chlorophyllide a oxygenase*
*CHI*: *Chalcone isomerase*
*CHLD*: *Magnesium chelatase subunit D*
*ChlG*: *Divinyl chlorophyllide a 8-vinyl-reductase*
*CHLH*: *Magnesium chelatasesubunit H*
*CHLI*: *Magnesium chelatasesubunit I*
*CHLM*: *Magnesium-protoporphyrin O-methyltransferase*
*CHS*: *Chalcone synthase*
*CLH*: *Chlorophyllase*
*CRD*: *Magnesium-protoporphyrin IX monomethyl ester (oxidative) cyclase (oxidative) cyclase*
DEG: Differentially expressed genes
*DFR*: *Dihydroflavonol 4-reductase*
*DVR*: *Divinyl chlorophyllide a 8-vinyl-reductase*
*F3’5’H*: *Flavonoid 3′,5′-hydroxylase*
*F3H*: *Naringenin, 2-oxoglutarate 3-dioxygenase*
FPKM: Fragments per kilobase of transcript per million mapped reads
*GluRS*: *Glutamate-tRNA ligase*
GO: Gene ontology
*GSA*: *Glutamate-1-semialdehyde 2,1-aminomutase*
*GST*: *Glutathione S-transferase*
*HCAR*: *7-hydroxymethyl chlorophyll a reductase*
*HEMA*: *Glutamyl-tRNA reductase*
*HEMC*: *Porphobilinogen deaminase*
*HEMD*: *Uroporphyrinogen-III synthase*
*HEME*: *Uroporphyrinogen decarboxylase*
*HEMF*: *Coproporphyrinogen III oxidase*
*HEMG*: *Oxygen-dependentprotoporphyrinogen oxidase*
*LDOX*: *Leucoanthocyanidin dioxygenase*
MYB: Myeloblastosis
NCBI: National Center for Biotechnology Information
*NYC1*: *Chlorophyll(ide) b reductase*
*PAL*: *Phenylalanine ammonia-lyase*
*PaO*: *Pheophorbide a oxygenase*
*POR*: *Protochlorophyllide reductase*
qRT-PCR: Quantitative reverse transcription polymerase chain reaction
*RCCR*: *Red chlorophyll catabolite reductase*
SRA: Sequence read achieve
TF: Transcription factor
*UGT*: *UDP-3-O-glucosyltransfersae*
WDR: WD repeat-containing protein
