# Supplementary material for: Transcriptome profiling of two contrasting ornamental cabbage (Brassica oleracea var. acephala) lines provides insights into purple and white inner leaf pigmentation
Source: BMC Genomics. 2018 Nov 6;19:797. doi: 10.1186/s12864-018-5199-3 (PMC6219265; doi:10.1186/s12864-018-5199-3)
Supplement: Supplementary file 6 — Figure S1. Expression pattern of MYB28.1, RL1 and MYBL2 using FPKM (fragment per kilobase of transcript per million mapped reads) values of ornamental cabbage leaf transcriptome. A, younger purple leaf; B, mid-age leaf (bicolor with purple at proximal end and green at distal end); C, older green leaf; D, younger white leaf; E, bicolor mid-age leaf (bicolor with white center and green margin); F, older light green leaf. (DOCX 144 kb) [file 12864_2018_5199_MOESM6_ESM.docx]

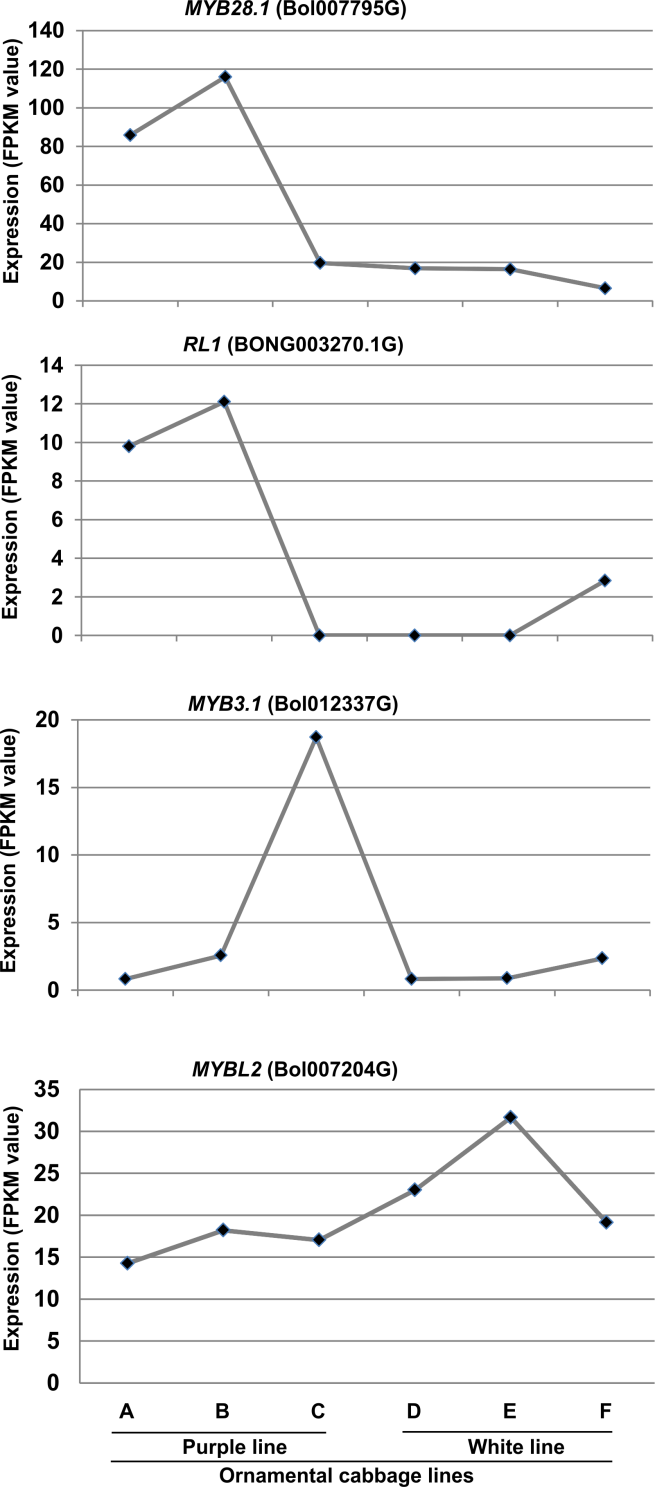


**Figure S1** Expression pattern of *MYB28.1, RL1* and *MYBL2* using FPKM (Fragment Per Kilobase of transcript per Million mapped reads) value of ornamental cabbage leaf transcriptome. A, younger purple leaf; B, mid-aged leaf (bicolor of purple at proximal end and green at distal end of the leaf) ; C, older green leaf; D, younger white leaf ; E, bicolor leaf (white center with green margin); F, light green leaf
